# Correlation of Inflammatory Parameters with the Development of Cerebral Vasospasm, Takotsubo Cardiomyopathy, and Functional Outcome after Spontaneous Subarachnoid Hemorrhage

**DOI:** 10.3390/jcm13071955

**Published:** 2024-03-28

**Authors:** Dorottya Szántó, Péter Luterán, Nikolett Kóti, Péter Siró, Éva Simon, Zsuzsa Jakab, Judit Gál, János Kappelmayer, Béla Fülesdi, Csilla Molnár

**Affiliations:** 1 Department of Anesthesiology and Intensive Care, Faculty of Medicine, University of Debrecen, 4032 Debrecen, Hungaryjakab.zsuzsanna@med.unideb.hu (Z.J.);; 2 Department of Laboratory Medicine, Faculty of Medicine, University of Debrecen, 4032 Debrecen, Hungary; 3 Hungarian Research Network (HUN-REN-DE) Cerebrovascular Research Group, 4032 Debrecen, Hungary

**Keywords:** subarachnoid hemorrhage, vasospasm, delayed cerebral ischemia, Takotsubo cardiomyopathy, systemic inflammatory response

## Abstract

**Background:** The present work aimed to determine whether a relationship exists between inflammatory parameters and the development of vasospasm (VS) and Takotsubo cardiomyopathy (TTC), as well as clinical outcome, in patients suffering from spontaneous subarachnoid hemorrhage (SAH). **Methods**: In this study, the authors processed the prospectively collected laboratory and clinical data of spontaneous SAH patients admitted to the neurointensive care unit between March 2015 and October 2023. The highest values of neutrophils (NEUpeak), monocytes (MONOpeak), neutrophil-to-lymphocyte ratio (NLRpeak), and CRP (CRPpeak) during the initial 7 days were correlated with the occurrence of VS and TTC, and with the outcome measures at day 30 after onset. **Results**: Data were collected from 175 SAH patients. Based on ROC analysis, for the development of VS, MONOpeak was the most accurate indicator (AUC: 0.619, optimal cut-off: 1.45 G/L). TTC with severe left ventricular dysfunction (ejection fraction < 40%) was indicated most sensitively by NEUpeak (ROC: 0.763, optimal cut-off: 12.34 G/L). Both for GOS and Barthel Index at day 30, CRPpeak was the best predictor for the outcome (GOS: ROC: 0.846, optimal cut-off: 78.33 mg/L and Barthel Index: ROC: 0.819, optimal cut-off: 78.33 mg/L). **Conclusions**: Laboratory parameters referring to inflammation during the initial 7 days after SAH correlate with the development of VS and TTC, and thus may predict functional outcome.

## 1. Introduction

The past decade has seen a meaningful improvement in the functional outcomes of patients presenting with aneurysmal subarachnoid hemorrhage (SAH). It is believed that the combination of the gradual advancement of endovascular treatment modalities and neurocritical care treatment options contributed most to this more favorable outcome. However, it has to be noted that roughly 10–12% of the cases die immediately before reaching the hospital; hospitalized patients still have high mortality and permanent disability rates; and roughly half of the survivors are unable to return to their previous lives [1,2]. In addition to early damage, the development of delayed cerebral ischemia (DCI) has a major effect on patients’ outcomes. Although the pathophysiological background of DCI is still not completely understood, cerebral vasospasm is one of the possible triggers [3].

In recent years, many experimental and clinical studies drew attention to inflammatory mechanisms that may play a determining role in the development of both early and delayed complications. After the insult, immune cells from the periphery are recruited into brain parenchyma and contribute to the release of inflammatory cytokines, resulting in the expansion of the injury [4]. Although the underlying mechanism of cerebral vasospasm is still not fully explored, there is growing evidence that neuroinflammation has a significant role [4,5,6]. Among widely available laboratory markers, leukocyte count, neutrophil-to-lymphocyte ratio, and C-reactive protein levels showed correlations with variable complications and poor functional outcomes [6,7,8,9,10,11,12].

SAH is the most common neurological cause of Takotsubo cardiomyopathy (TTC). TTC is an acute, reversible cardiomyopathy that can be triggered by a variety of emotional or physical stressors. The prevalence of TTC in SAH ranges from 1.2% up to 28%, and it is associated with higher mortality and poor functional outcomes. The pathomechanism of TTC is not completely understood, but catecholamine-induced myocardium stunning seems to have a prominent role. There is also growing evidence for the pathogenetic role of inflammatory mechanisms, reflected by generally elevated C-reactive protein in TTC [13,14]. Recent studies revealed that systemic inflammation might have important pathogenic and prognostic implications in the general TTC patient population [15]; however, data on SAH-associated TTC are lacking.

Based on all these factors, in this present study, the authors aimed to reveal the correlation between commonly used inflammatory markers and the development and severity of cerebral vasospasm and Takotsubo cardiomyopathy after spontaneous SAH. In addition, the secondary aim of this study was to evaluate the predictive value of the studied inflammatory parameters for patients’ outcomes.

## 2. Materials and Methods

The authors of this article performed a secondary analysis of the data of their two previous prospective studies (Clinical Trials Registration Numbers: NCT02659878, NCT05403970; ethic approval dates: 26 February 2015, and 15 December 2021) that were conducted at the 8-bed neurosurgical ICU of the Department of Anesthesiology and Intensive Care, University of Debrecen.

Inclusion criteria in both studies were acute non-traumatic subarachnoid hemorrhage (aneurysmal SAH, aneurysmal appearing SAH without identified aneurysm or perimesencephalic SAH) in adult patients (age > 18 years) and hospital admission within 48 h after symptom onset. Patients with head trauma, angioma, or arteriovenous malformations were excluded. All included patients or their closest relatives gave written informed consent before participation.

The World Federation of Neurological Society (WFNS) score, Hunt–Hess score, and modified Fisher grade were recorded at admission. For this analysis, the following laboratory tests were processed, which were performed at admission and repeated every second day during the first week after the onset of symptoms: blood neutrophil count (NEU), blood monocyte count (MONO), blood lymphocyte count, and C-reactive protein level (CRP). Additionally, the neutrophil-to-lymphocyte ratio (NLR) was calculated.

The development of vasospasm was monitored with transcranial color-coded Duplex (TCCD) sonography. TCCD sonography was performed by two experienced investigators (BF, PS) using the 2 MHz sector transducer of the GE Venue Go (GE Healthcare 9900, Innovation Drive, Wauwatosa, WI, USA) ultrasound device. The investigators were unaware of the clinical data, but knew that the patient suffered from SAH. The transtemporal window was used for insonation. After identifying the different vessels of the circle of Willis, daily measurements of the middle cerebral, anterior cerebral, and posterior cerebral arteries were performed on both sides. Cerebral blood flow velocities, pulsatility indices, and S/D ratios (systolic velocity/diastolic velocity) were documented in all cases. Based on previous suggestions [16], vasospasm was considered if the mean blood flow velocity was higher than 120 cm/s, and severe vasospasm was diagnosed if the mean blood flow velocity exceeded 200 cm/s.

For this secondary analysis, clinical data on the presence or absence of Takotsubo cardiomyopathy were also processed, if available. The diagnosis of Takotsubo cardiomyopathy was based on the Modified Mayo criteria, taking the following aspects into account:

- Transient left/right ventricle wall motion abnormalities, usually extending beyond a single epicardial coronary artery distribution;
- Evidence of myocardial involvement by biomarker elevation: cardiac troponin I, creatine kinase, brain natriuretic peptide, N-terminal prohormone of brain natriuretic peptides;
- ECG abnormalities: ST elevation/depression, negative T-waves, new bundle branch blocks;
- Potential coronary artery culprit ruled out [17].

Transthoracic echocardiography was performed at admission and was repeated on day 7, day 30, and day 180. Electrocardiography (ECG) and cardiac biomarkers (cardiac troponin T, creatine kinase, N-terminal prohormone of brain natriuretic peptide) were checked daily during the first week. If new ECG abnormalities or cardiac biomarker elevation were detected between days 1 and 7, additional echocardiography was performed immediately. Indications for further diagnostic steps (coronary computed tomography or coronary angiography) were established by cardiologists in each case. Based on the lowest ejection fraction measured during the acute phase, we distinguished mild (EF ≥ 40%) and severe TTC (EF < 40%) groups.

Mortality data were recorded and Glasgow Outcome Scale and Barthel Index assessments were performed during the 30-day follow-up visit.

For statistical analysis, the SPSS 29.0 (IBM Corp., Armonk, NY, USA) statistical software was used. The peak values of NEU (NEUpeak), MONO (MONOpeak), NLR (NLRpeak), and CRP (CRPpeak) measured during the first week after SAH were correlated with the severity of SAH, the development of cerebral vasospasm, the development of TTC, and outcome measures. The homogeneity of variances was checked with Levene’s test, and the normal distribution of variables was checked by the Shapiro–Wilk test. All studied variables were non-normally distributed and/or had heterogeneous variances. Descriptive statistics were thus reported as medians (IQR). The Mann–Whitney U-test was used to compare the differences between two independent groups. For multiple variables, the Kruskal–Wallis test was performed. If the null hypothesis of Kruskal–Wallis’s test was rejected, post hoc pairwise analyses were performed with Dunn–Bonferroni’s test. Receiver operating characteristic (ROC) curve analysis was performed to evaluate the performance of laboratory tests to indicate the development of sonographic vasospasm and Takotsubo cardiomyopathy, as well as to predict outcomes. The area under the curve and 95% confidence intervals were calculated. *p* < 0.05 was considered statistically significant.

## 3. Results

The present analysis included a total of 175 adult patients with acute spontaneous SAH. There were 106/175 (60.57%) women and 69/175 (39.43%) men, with an average age of 55.34 ± 10.97 (mean ± SD). The most important clinical data of the patients are summarized in Table 1.

### 3.1. The Relationship between Inflammatory Markers and SAH Severity at Admission

Significantly higher NEU, NLR, MONO, and CRP peaks were related to higher modified Fisher’s grade SAH, as shown in Table 2. Similar to Fisher’s grade, the more severe the neurological status of the patient was at admission, the higher the observed NEUpeak, NLRpeak, MONOpeak, and CRPpeak values were. The corresponding values are summarized in Table 2.

### 3.2. Relationship between Inflammatory Markers and the Development of Cerebral Vasospasm

In the entire cohort, 140/175 patients had an adequate acoustic bone window for transcranial color-coded Duplex sonography. Sonographic vasospasm occurred in 68/140 (48.57%) cases; among them, 25/68 (17.86%) were severe. The comparison of the different inflammatory parameters between the vasospasm and the non-vasospasm group is summarized in Table 3. NEUpeak, NLRpeak, and MONOpeak values were significantly higher in the vasospasm (VS) than in non-vasospasm (nVS) groups, whereas the difference for CRP did not reach the level of statistical significance.

We also performed a secondary analysis by forming subgroups of severe (sVS) and moderate vasospasm (mVS). It has been found that there is a significant difference in NEUpeak and MONOpeak values depending on the severity of vasospasm. Significantly higher NEUpeak values were demonstrated in patients with severe vasospasm versus patients without vasospasm (nVS). The moderate VS group did not significantly differ from the nVS or sVS group. For MONOpeak, significantly higher values were related to severe vasospasm compared to the mVS or nVS group; however, there was no significant difference between the nVS and mVS groups (Table 4).

**Figure 1:**
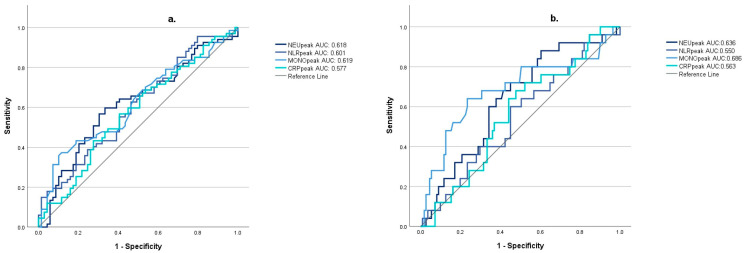
The diagnostic accuracy of inflammatory markers for sonographic vasospasm (**a**) and severe sonographic vasospasm (**b**). (NEUpeak: peak neutrophil count during the first week after subarachnoid hemorrhage; NLRpeak: peak neutrophil-to-lymphocyte ratio during the first week after subarachnoid hemorrhage; MONOpeak: peak monocyte count during the first week after subarachnoid hemorrhage; CRPpeak: peak C-reactive protein value during the first week after subarachnoid hemorrhage).

MONOpeak was the most accurate indicator of any type of sonographic vasospasm (VS) (AUC: 0.619 (95% CI: [0.524; 0.714]; optimal cutoff value: 1.45), and also of severe sonographic vasospasm (sVS) (AUC: 0.686 (95% CI: [0.554; 0.818]; optimal cutoff value: 1.33) (Figure 1).

### 3.3. Correlation of Inflammatory Markers with the Severity of Takotsubo Cardiomyopathy

In total, 121/175 patients had available serial echocardiography reports. Additionally, 38/121 (31.4%) had confirmed TTC, of which 11/121 (9.1%) were associated with severe left ventricular dysfunction (left ventricular ejection fraction < 40%). For each studied inflammatory marker, the difference was significant between the non-TTC, mild TTC, and severe TTC groups, mostly because of the high values in the severe TTC group (Table 5).

**Figure 2:**
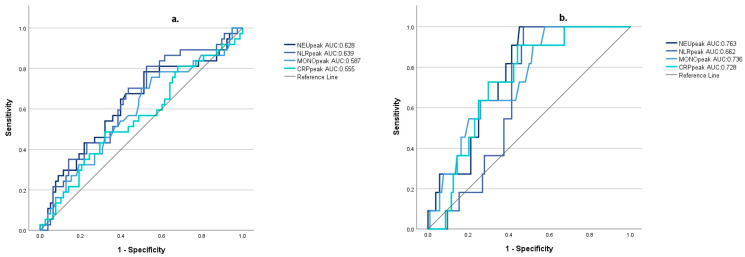
The diagnostic accuracy of inflammatory markers for TTC (with or without severe left ventricular dysfunction) (**a**) and TTC with severe left ventricular dysfunction (left ventricular ejection fraction < 40%); (**b**) (NEUpeak: peak neutrophil count during the first week after subarachnoid hemorrhage; NLRpeak: peak neutrophil-to-lymphocyte ratio during the first week after subarachnoid hemorrhage; MONOpeak: peak monocyte count during the first week after subarachnoid hemorrhage; CRPpeak: peak C-reactive protein value during the first week after subarachnoid hemorrhage).

Based on ROC analysis results, NEUpeak was the most accurate indicator of the development of TCC with severe left ventricular dysfunction (AUC: 0.763; 95% CI: [0.656; 0.869]; optimal cutoff value: 12.34) (Figure 2).

### 3.4. The Predictive Value of Inflammatory Markers on Patient Outcome

Higher 1-week mortality and unfavorable 30-day outcomes, according to the Glasgow Outcome Scale (GOS score < 4) and Barthel Index (BI < 50), were associated with higher values of the studied inflammatory markers. All the differences were significant (Table 6).

**Figure 3:**
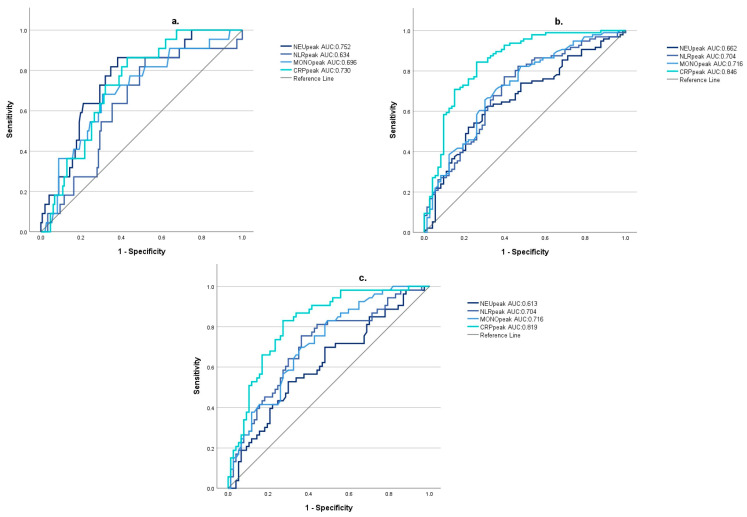
The predictive value of inflammatory markers for 1-week survival (**a**), 30-day Glasgow Outcome Scale score < 4 (**b**), and 30-day Barthels’ Index < 50 (**c**) (NEUpeak: peak neutrophil count during the first week after subarachnoid hemorrhage; NLRpeak: peak neutrophil-to-lymphocyte ratio during the first week after subarachnoid hemorrhage; MONOpeak: peak monocyte count during the first week after subarachnoid hemorrhage; CRPpeak: peak C-reactive protein value during the first week after subarachnoid hemorrhage).

For 1-week mortality ROC analysis indicated the best predictive value of NEUpeak (AUC:0.752; 95% CI [0.652; 0.852]; optimal cutoff value: 12.87). For unfavorable 30-day outcomes, defined based on GOS and Barthel Index, CRPpeak was the most sensitive predictor (GOS AUC: 0.846; 95% CI [0.784; 0.907]; optimal cutoff value: 78.33; BI AUC: 0.819, 95% CI [0.746; 0.891], optimal cutoff value: 78.33) (Figure 3).

## 4. Discussion

Spontaneous subarachnoid hemorrhage is characterized by a mortality rate of up to 35%. More than 50% of survivors make an incomplete recovery, and approximately one-third of the survivors remain severely disabled and functionally dependent [18]. In addition to the injury occurring in the ictal phase, secondary damage triggered by cerebral vasospasm and delayed cerebral ischemia has a determining effect on the outcome. Based on experimental and clinical research, there is growing evidence that neuroinflammation plays a pivotal role in both early and delayed injury [4,19,20,21]. Studies have shown that, in up to 60% of patients with hemorrhagic stroke, a systemic inflammatory response syndrome may develop [8]. As a consequence, the predictive value of inflammatory markers and the efficiency of anti-inflammatory therapy has become a central issue [1,22]. Based on this, in the present study, we attempted to reveal the prognostic value of widely available and routinely checked inflammatory markers (NEU, NLR, MONO, CRP), with the intention that this may facilitate the early identification and adequate management of patients at higher risk of complications and poor outcomes.

As is consistent with previous research [9,19,23], all studied inflammatory markers reached significantly higher peak values within the first week in patients with higher modified Fisher’s grades and patients with poor initial neurological states (Hunt–Hess IV-V). This reflects an enhanced systemic inflammatory response, which is, according to the present knowledge, mediated by increased catecholamine release at the ictus, as well as increased cytokine release induced by necrotic and apoptotic neurons and the degrading hematoma [4,19,24].

Despite the thorough research in the past decades, VS remains an unpredictable and poorly understood complication of SAH. The development of VS notably increases the risk of disability; therefore, early identification of high-risk patients is of the utmost importance. There is growing evidence that the inflammatory response plays a prominent role in the pathogenesis of VS; consequently, previous studies have also attempted to investigate the predictive value of certain inflammatory markers. Recently, in a retrospective study, Buce-Satoba et al. demonstrated a relationship between white blood cell count and CRP values at admission and the development of VS [6]. Another retrospective study by Kula et al. found significantly higher NLR in VS patients on days 6–10 and 12–13 after the insult [7]. In addition, several clinical studies have demonstrated associations between the appearance of DCI and higher values of inflammatory markers [25,26,27,28,29,30]. In the present study, we found a significant correlation between NEUpeak, NLRpeak, and MONOpeak and the development of VS; furthermore, MONOpeak showed a correlation with the severity of vasospasm. However, all studied inflammatory markers have shown poor accuracy in indicating cerebral vasospasm. Thus, their use may serve as a complementary prognostic tool, along with widely used clinical and radiological classification scores, and may facilitate the identification of high-risk patients.

The present report is among the first studies to analyze the relationship between systemic inflammatory response and the development and severity of SAH-associated TTC based on prospectively collected data. TTC is a relatively common phenomenon occurring in patients with SAH and may have a major effect on cerebral circulation and predict poor outcomes. In our cohort, 121/175 patients had available serial echocardiography reports, and TTC of any severity was confirmed in 31,4% of the cases. We arbitrarily defined a mild and a severe group of TTC patients based on ejection fraction. The predefined threshold of 40% ejection fraction (EF) was based on previous reports indicating that the critical value of EF that determines cardiovascular complications lies between 40 and 45%. Catecholamine release triggered by the ictus plays a central role in the pathophysiology of TTC [14]. Catecholamines also trigger peripheral immune cell recruitment; thus, higher immune cell peaks may reflect a more intense catecholamine surge and may draw attention to the higher risk of TTC. In line with this theory, in our study, the NEU, NLR, MONO, and CRP peaks significantly correlated with the development of severe TTC, and the NEU, MONO, and CRP peaks also showed significant differences between severe TTC, moderate TTC, and the control groups as well. All the studied inflammatory markers were found to be poor indicators for TTC; however, for severe TTC, NEUpeak appeared to have a moderate accuracy. Similarly to our findings, retrospective studies by Geraghty et al. and Zhang et al. demonstrated that the total leukocyte, neutrophil, and monocyte counts were independent predictors of reduced left ventricular systolic function after SAH [31,32]. According to recent studies, the systemic inflammatory response not only coincides with, but may also contribute to, the development of TTC. However, our data are insufficient to prove this concept [15].

All studied inflammatory parameters significantly correlated with 1-week mortality and also with 30-day outcome measures. For 1-week mortality, NEUpeak and CRPpeak had moderate predictive value. NLRpeak and MONOpeak were moderate, and CRPpeak was a good predictor for the 30-day outcome according to GOS and BI. In line with our results, Alessandro et al. also found a positive correlation between early CRP elevation and worse neurological outcomes at hospital discharge [31]. In addition, a large, retrospective analysis including 1017 patients indicated that CRP is an independent predictor of outcome after SAH [33]. Furthermore, several retrospective studies demonstrated that elevated NLR is an independent predictor of poor outcomes [8,9]. Unda et al. also demonstrated a positive correlation between admission monocyte count and poor functional outcomes at discharge and 12-month follow-up [30].

We have to mention some limitations of our study, as well. First, as a single-center study, the number of included patients was limited. Second, we did not have sufficient data to be able to separately analyze the clinically more relevant symptomatic vasospasm group. Furthermore, for the same reason, we were not able to report whether inflammatory markers were also associated with the development of DCI. Third, the development of pneumonia, sepsis, meningitis, or other infections may have a major influence on inflammatory parameters and may also affect the outcome. Although our database did not include clinical diagnoses indicating infections, procalcitonin (PCT) levels above 2 ng/mL (which may indicate a severe bacterial infection) were significantly more common among patients with worse outcomes (GOS 1-3 vs. GOS 4-5: 12.24% vs. 0%, Fisher’s exact test, *p* = 0.002) and might have affected our results. In contrast, the development of vasospasm and TTC did not show an association with PCT > 2 ng/mL. Fourth, we only evaluated the prognosis of patients 30 days after the hemorrhage, and long-term follow-up data may be warranted to draw stronger conclusions on the findings. Fifth, due to the small sample size, we were not able to perform multivariate logistic regression to describe the effects of possible confounders.

## 5. Conclusions

In conclusion, our findings confirm the previous results that early CRP elevation is a good predictor of poor neurological outcomes after SAH. Although all of the investigated inflammatory markers were found to be poor or moderate indicators of VS or TTC, their simplicity and wide availability may make them useful additional tools in daily clinical practice. Further studies are needed to clarify the relationship between inflammatory response and SAH-associated TTC.

## Figures and Tables

**Table 1:** Characteristics and confounding factors in the entire cohort.

Parameter	Value
**Age (years, mean ± SD)**	55.34 ± 10.97
**Gender (F/M)**	69/106
**Location of the aneurysm**	
Internal carotid	23
Middle cerebral	40
Anterior cerebral and anterior communicating	57
Posterior cerebral and posterior communicating	8
Basilar and vertebral	13
Other	9
Without identified aneurysm	25
**Modified Fisher grade at admission**	
I–II	56
III–IV	119
**Hunt–Hess score at admission**	
I–III	109
IV–V	66
**Vasospasm**	
Moderate	43
Severe	25
Without vasospasm	72
No data available	35
**Takotsubo cardiomyopathy (TTC)**	
TTC with moderate left ventricular dysfunction	27
TTC with severe left ventricular dysfunction	14
Without TTC	86
No data available	48
**Mortality**	
1-week mortality	22
30-day mortality	39
**30-day Glasgow outcome scale score**	
1–3	99
4–5	74
No data available	2
**30-day Barthel Index (survivors)**	
<50	53
50≤	81
No data available	2

**Table 2:** The serum concentration of inflammatory markers in groups according to the severity of subarachnoid hemorrhage at admission (NEUpeak: peak neutrophil count during the first week after subarachnoid hemorrhage; NLRpeak: peak neutrophil-to-lymphocyte ratio during the first week after subarachnoid hemorrhage; MONOpeak: peak monocyte count during the first week after subarachnoid hemorrhage; CRPpeak: peak C-reactive protein value during the first week after subarachnoid hemorrhage).

**Modified Fisher’s Grade at Admission**			
**Inflammatory Markers**	**Modified Fisher I–II**	**Modified Fisher III–IV**	**Mann–Whitney U-Test**
NEUpeak	10.29 (IQR 5.87)	12.87 (IQR 6.37)	*p* = 0.005 *
NLRpeak	8.29 (IQR 9.94)	13.28 (IQR 11.49)	*p* < 0.001 *
MONOpeak	0.85 (IQR 0.74)	1.1 (IQR 0.6)	*p* = 0.003 *
CRPpeak	30.41 (IQR 74.25)	155.1 (IQR 171.91)	*p* < 0.001 *
**Hunt–Hess grading at admission**			
**Inflammatory markers**	**Hunt–Hess I–III**	**Hunt–Hess IV–V**	**Mann–Whitney U-test**
NEUpeak	10.81 (IQR 5.31)	14.9 (IQR 6.45)	*p* < 0.001 *
NLRpeak	9.48 (IQR 10.56)	14.27 (IQR 9.63)	*p* < 0.001 *
MONOpeak	0.95 (IQR 0.61)	1.27 (IQR 0.57	*p* < 0.001 *
CRPpeak	48.85 (IQR 138.95)	195.3 (IQR 153.56)	*p* < 0.001 *

* signs statistically significant difference.

**Table 3:** The serum concentration of inflammatory markers in groups according to the occurrence of vasospasm (VS) (NEUpeak: peak neutrophil count during the first week after subarachnoid hemorrhage; NLRpeak: peak neutrophil-to-lymphocyte ratio during the first week after subarachnoid hemorrhage; MONOpeak: peak monocyte count during the first week after subarachnoid hemorrhage; CRPpeak: peak C-reactive protein value during the first week after subarachnoid hemorrhage).

Inflammatory Markers	Non-Vasospasm (nVS) Group	Vasospasm (VS) Group	Mann–Whitney U-Test
NEUpeak	11.24 (IQR 5.15)	13.42 (IQR 7.34)	*p* = 0.018 *
NLRpeak	9.69 (IQR 9.82)	13.24 (IQR 12.48)	*p* = 0.042 *
MONOpeak	0.95 (IQR 0.53)	1.05 (IQR 0.71)	*p* = 0.016 *
CRPpeak	74.6 (IQR 152.7)	110.56 (IQR 183.81)	*p* = 0.077

* signs statistically significant difference.

**Table 4:** The serum concentration of inflammatory markers in groups according to the severity of vasospasm (VS) (NEUpeak: peak neutrophil count during the first week after subarachnoid hemorrhage; NLRpeak: peak neutrophil-to-lymphocyte ratio during the first week after subarachnoid hemorrhage; MONOpeak: peak monocyte count during the first week after subarachnoid hemorrhage; CRPpeak: peak C-reactive protein value during the first week after subarachnoid hemorrhage).

Inflammatory Markers	Non-Vasospasm (nVS) Group	Mild Vasospasm (mVS) Group	Severe Vasospasm (sVS) Group	Kruskal–Wallis (+ Dunn–Bonferroni’s Test)
NEUpeak	11.24 (IQR 5.15)	12.61 (IQR 7.66)	13.79 (IQR 5.88)	*p* = 0.032 * (nVS vs. sVS *p* = 0.012 * mVS vs. sVS *p* = 0.262 nVS vs. mVS *p* = 0.125)
NLRpeak	9.69 (IQR 9.82)	13.48 (IQR 12.89)	13.01 (IQR 11.18)	*p* = 0.123
MONOpeak	0.95 (IQR 0.53)	1.0 (IQR 0.63)	1.47 (IQR 0.72)	*p* = 0.008 * (nVS vs. sVS *p* = 0.002 * mVS vs. sVS *p*= 0.047 * nVS vs. mVS *p* = 0.251)
CRPpeak	74.6 (IQR 152.7)	103.75 (IQR 193.48)	130.09 (IQR 164.71)	*p* = 0.203

* signs statistically significant difference.

**Table 5:** The serum concentration of inflammatory markers in groups according to the severity of Takotsubo cardiomyopathy (TTC) (NEUpeak: peak neutrophil count during the first week after subarachnoid hemorrhage; NLRpeak: peak neutrophil-to-lymphocyte ratio during the first week after subarachnoid hemorrhage; MONOpeak: peak monocyte count during the first week after subarachnoid hemorrhage; CRPpeak: peak C-reactive protein value during the first week after subarachnoid hemorrhage).

Inflammatory Markers	Non-TTC Group	Mild TTC Group	Severe TTC Group	Kruskal–Wallis (+ Dunn–Bonferroni’s Test)
NEUpeak	12.04 (IQR 6.87)	12.47 (IQR 8.95)	16.28 (IQR 5.37)	*p* = 0.011 * (nTTC vs. sTTC *p* = 0.003 * mTTC vs. sTTC *p* = 0.031 * nTTC vs. mTTC *p* = 0.391)
NLRpeak	9.56 (IQR 10.22)	11.62 (IQR 14.65)	14.93 (IQR 4.28)	*p* = 0.046 * (nTTC vs. sTTC *p*= 0.038 * mTTC vs. sTTC *p* = 0.427 nTTC vs. mTTC *p* = 0.086)
MONOpeak	0.94 (IQR 0.61)	1.05 (IQR 0.52)	1.39 (IQR 0.65)	*p* = 0.035 * (nTTC vs. sTTC *p* = 0.01 * mTTC vs. sTTC *p* = 0.039 * nTTC vs. mTTC *p* = 0.668)
CRPpeak	58.45 (IQR 191.75)	84.1 (IQR 169.63)	215.61 (IQR 122.15)	*p* = 0.036 * (nTTC vs. sTTC *p*= 0.012 * mTTC vs. sTTC *p* = 0.019 * nTTC vs. mTTC *p* = 0.875)

* signs statistically significant difference.

**Table 6:** Inflammatory parameters and outcome (NEUpeak: peak neutrophil count during the first week after subarachnoid hemorrhage; NLRpeak: peak neutrophil-to-lymphocyte ratio during the first week after subarachnoid hemorrhage; MONOpeak: peak monocyte count during the first week after subarachnoid hemorrhage; CRPpeak: peak C-reactive protein value during the first week after subarachnoid hemorrhage).

**1-Week Survival**			
Inflammatory markers	Survival > 1 week	Survival ≤ 1 week	Mann–Whitney U-test
NEUpeak	11.76 (IQR 6.04)	16.25 (IQR 4.03)	*p* < 0.001 *
NLRpeak	10.58 (IQR 10.47)	16.49 (IQR 8.24)	*p* = 0.041 *
MONOpeak	1.03 (IQR 0.64)	1.44 (IQR 0.64)	*p* = 0.003 *
CRPpeak	84.75 (IQR 179.15)	206.31 (IQR 141.34)	*p* < 0.001 *
**Glasgow outcome scale (GOS) at day 30**			
Inflammatory markers	GOS ≥ 4	GOS < 4	Mann–Whitney U-test
NEUpeak	10.81 (IQR 5.37)	14.1 (IQR 6.95)	*p* < 0.001 *
NLRpeak	8.33 (IQR 8.58)	14.27 (IQR 11.18)	*p* < 0.001 *
MONOpeak	0.88 (IQR 0.65)	1.2 (IQR 0.63)	*p* < 0.001 *
CRPpeak	27.3 (IQR 72.4)	186.5 (IQR 167.3)	*p* < 0.001 *
**Barthel Index (BI) at day 30**			
Inflammatory markers	BI ≥ 50	BI < 50	Mann–Whitney U-test
NEUpeak	10.81 (IQR 5.26)	12.45 (IQR 6.13)	*p* = 0.029 *
NLRpeak	8.33 (IQR 8.07)	15.12 (IQR 10.87)	*p* < 0.001 *
MONOpeak	0.88 (IQR 0.6)	1.2 (IQR 0.63)	*p* < 0.001 *
CRPpeak	34.32 (IQR 74.0)	167.55 (IQR 142.27)	*p* < 0.001 *

* signs statistically significant difference.

## Data Availability

Data relating to this article are available from the corresponding author upon reasonable request.
